# Length of stay and reason for admission in an adolescents inpatient unit

**DOI:** 10.1192/j.eurpsy.2022.1131

**Published:** 2022-09-01

**Authors:** M. Taracena Cuerda, M. Esperesate Pajares, M. Feito Garcia, C. Arranz Martin, E. Sánchez Sampedro, A.M. Jiménez Bidón, R. Puente García, C. Pastor Jordá

**Affiliations:** Hospital 12 de Octubre, Psychiatry, Madrid, Spain

**Keywords:** Adolescents, inpatient unit, Length os stay, reason for admission

## Abstract

**Introduction:**

Psychiatric Inpatient units are important resources of the mental health network. These units have elevated costs, so it is important to get to know some factos that might mediate the lengh of stay in these units.

**Objectives:**

Psychiatric Inpatient units are important resources of the mental health network. These units have elevated costs, so it is important to get to know some factos that might mediate the lengh of stay in these units.

**Methods:**

An observational and descriptive analysis of the sample of patients between 12 and 17 years-old, that were admitted to the inpatient mental health unit since its opening on April 2021.

**Results:**

205 patients were admitted April 2021 until October 2021. The most common reason for admission (RFA) was suicidal ideation/attempt (57.07%), eating disorders (15.1%), mood disorders (11.2%), conduct disorders/challenging behaviors (7.8%) and psychosis (7.3%). Adolescents with eating disorders had the longest length of stay, with an average of 23.8 days. They were followed by those suffering from psychosis (17.8 days) and suicidal ideation/attempts (17.1 days). Mood disorders average length of stay was 15.1 days and conduct disorders/challenging behaviors was the shortest one with a LOS of 12.5 days.

**Conclusions:**

Adolescents with eating disorders seem to need longer lentgh of stay, what differs from Zeshan et al study that concludes that patients with schizophrenia might need longer LOS. Nevertheless, just as Zeshan et al study, we conclude that patients admitted with conduct disorders/challenging behaviors have the shortest LOS.

**Disclosure:**

No significant relationships.

